# Experimental demonstration of coupled differential oscillator networks for versatile applications

**DOI:** 10.3389/fnins.2023.1294954

**Published:** 2023-12-04

**Authors:** Manuel Jiménez, Juan Núñez, Jafar Shamsi, Bernabé Linares-Barranco, María J. Avedillo

**Affiliations:** ^1^Instituto de Microelectrónica de Sevilla, IMSE-CNM (CSIC/Universidad de Sevilla), Seville, Spain; ^2^Department of Cell Biology and Anatomy, University of Calgary, Calgary, AB, Canada

**Keywords:** oscillatory neural networks, nano-oscillators, ASIC, phase-change material, neuromorphics, integrated circuits

## Abstract

Oscillatory neural networks (ONNs) exhibit a high potential for energy-efficient computing. In ONNs, neurons are implemented with oscillators and synapses with resistive and/or capacitive coupling between pairs of oscillators. Computing is carried out on the basis of the rich, complex, non-linear synchronization dynamics of a system of coupled oscillators. The exploited synchronization phenomena in ONNs are an example of fully parallel collective computing. A fast system’s convergence to stable states, which correspond to the desired processed information, enables an energy-efficient solution if small area and low-power oscillators are used, specifically when they are built on the basis of the hysteresis exhibited by phase-transition materials such as VO_2_. In recent years, there have been numerous studies on ONNs using VO_2_. Most of them report simulation results. Although in some cases experimental results are also shown, they do not implement the design techniques that other works on electrical simulations report that allow to improve the behavior of the ONNs. Experimental validation of these approaches is necessary. Therefore, in this study, we describe an ONN realized in a commercial CMOS technology in which the oscillators are built using a circuit that we have developed to emulate the VO_2_ device. The purpose is to be able to study in-depth the synchronization dynamics of relaxation oscillators similar to those that can be performed with VO_2_ devices. The fabricated circuit is very flexible. It allows programming the synapses to implement different ONNs, calibrating the frequency of the oscillators, or controlling their initialization. It uses differential oscillators and resistive synapses, equivalent to the use of memristors. In this article, the designed and fabricated circuits are described in detail, and experimental results are shown. Specifically, its satisfactory operation as an associative memory is demonstrated. The experiments carried out allow us to conclude that the ONN must be operated according to the type of computational task to be solved, and guidelines are extracted in this regard.

## 1 Introduction

Current society demands more and more applications that require applying computationally hard and data-intensive algorithms, for example, neural networks. These are generally run on devices such as CPUs or GPUs, which offer great computing power but also require high energy consumption for their operation, which limits their use in edge computing. An alternative to the use of CPUs or GPUs is their implementation in hardware. Currently, the development of these custom-specific hardware platforms is an area of high interest. It comprises many approaches, including digital and analog implementations. In the latter, the use of unconventional computing devices and paradigms is very promising. In this line of oscillatory neural networks (ONNs), the connection of a multitude of oscillator circuits by means of electrical coupling elements creates an intelligent collective system called oscillatory neural networks (ONNs) (Hoppensteadt and Izhikevich, 1999, 2000; Follmann et al., 2015; Sharma et al., 2015; Raychowdhury et al., 2019), with a high potential for energy-efficient computing. In ONNs, neurons are implemented with oscillators and synapses with resistive and/or capacitive coupling between pairs of oscillators. Computing is carried out on the basis of the rich, complex, non-linear synchronization dynamics of a system of coupled oscillators. When the oscillators synchronize in frequency, they tend to adopt a phase relationship that minimizes energy. The most commonly used ONN encodes information about the relationship between oscillator phases. Depending on the type of coupling, the phases of two interconnected oscillators tend to get closer (to be in phase) or to separate (to be out of phase or anti-phase). The energy landscape of the system is determined by the coupling configuration. Thus, the idea behind computing with ONNs is to map the solutions of the target task into their minimal energy states. The exploited synchronization phenomena are an example of what is called collective computing and are fully parallel. Convergence to the stable system state is fast, which paves the way for energy efficiency associated with low computation times. It has been proposed to be used as associative memory (AM) by configuring the couplings such that the patterns to be stored (training patterns) are minimal energy states of the system (Nikonov et al., 2015). ONNs are also useful for solving optimization problems by formulating them as an Ising model (Lucas, 2014) and mapping them to an ONN (Dutta et al., 2021). The Ising model problem is solved by the natural evolution of the ONN state to states associated with minimum values in its energy function (Hamiltonian). The relationship between ONNs and Hopfield neural networks (HNNs) (Hopfield, 1982) is evident at this point.

ONN implementations with different types of oscillators have been reported (phase-locked loops and voltage-controlled oscillators (Hoppensteadt and Izhikevich, 2000), non-volatile logic based on magnetic tunnel junctions (Calayir and Pileggi, 2013), micro-electro-mechanical systems and a feedback loop with transconductance amplifiers (Kumar and Mohanty, 2017), comparator and a digital circuit in Jackson et al. (2018), CMOS ring oscillators (Csaba et al., 2016; Ahmed et al., 2021; Moy et al., 2022), STOs (Popescu et al., 2018), or VO_2_ (Corti et al., 2018), (Dutta et al., 2019, 2021; Corti et al., 2020; Núñez et al., 2021).

Structurally, the ONN resembles an artificial network based on the Hopfield model, HNN, which has been studied in-depth with regard to AM and pattern recognition tasks. The HNN has a simple conceptual model comprising a single, recurrent, fully connected layer of neurons with synaptic weights. Typically, the HNN model considers bipolar-state neurons. The state of each neuron is represented by $S_{i}$ and it takes values in {−1, +1}. State updates as:


(1) $$S_{i}=\mathrm{sign}\;\left(\underset{j}{\sum }W_{ij}S_{j}\right)$$

with *W*_ij_ the weight of the synapse connecting neuron *i* and neuron *j*, *W*_ij_ = *W*_ji_, and *W*_ii_ = 0. The HNN gradually transitions from an initial input state until a fixed point is reached. Fixed (stable or attractors) states are determined by the synaptic weights. In AM applications, the weight values are assigned (trained) in such a way that the patterns to be stored are fixed as attractor states. When an input pattern is applied, it evolves toward the closest stored pattern. In other words, when a distorted version of a training pattern is applied to the HNN, the original one is retrieved (inference).

Of course, energy-efficient oscillators are also necessary to achieve the target goal of energy-efficient computation. In this sense, oscillatory-based computing is not new. There were early contributions from pioneers such as Von Neumann (1957) and Goto (1959) in the 1950s. However, recent advances in technology have made it a popular and active research area. This is due to the emergence of phase-transition devices that can implement highly efficient and compact oscillators with minimal energy consumption based on various physical phenomena. VO_2_ devices, in particular, stand out for their hysteresis in the characteristic I–V curve, which enables compact low-power relaxation oscillators (Csaba and Porod, 2020).

**Figure 1:**
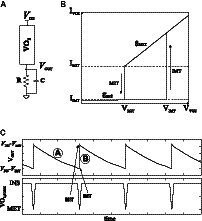
**(A)** I–V characteristic of the VO_2_ device. **(B)** VO_2_-based oscillator. **(C)** Output waveform of the oscillator including the state of the VO_2_ device.

VO_2_ material undergoes metal–insulator transitions under given electrical stimuli. That is, abrupt switching occurs from/to a high resistivity state (insulating phase) to/from a low resistivity state (metallic phase). Without electrical stimuli, it tends to stabilize in the insulating phase. When the applied voltage increases and the current density flowing through it reaches a given amount, an insulator-to-metal transition (IMT) occurs. Once in the metallic state, when the voltage decreases and the current density drops below a second given value, a metal-to-insulator transition (MIT) takes place. Figure 1A shows the I–V characteristic of a generic VO_2_. A compact oscillator has been proposed on its basis (Figure 1B; Maffezzoni et al., 2015; Parihar et al., 2015). Figure 1C depicts waveforms for the oscillator output. The state of the VO_2_ is also shown to better illustrate the circuit behavior. VO_2_,_STATE_ = ‘INS’ means the device is in the insulating state. VO_2,STATE_ *=* ‘MET’ corresponds to the device in the metallic state. Assuming that the VO_2_ is in an insulating state (marked with “A” in Figure 1C), the oscillator output is discharged through the resistor. This increases the voltage drop across the VO_2_ (*V*_DD_ – *V*_OUT_) and so does the current through it. Once enough current density circulates, it switches to the metallic state (marked with “B” in Figure 1C). Equivalently, using the electrical model, switching to the metallic state occurs once the VO_2_ voltage reaches *V*_IMT_. The capacitor is then charged through the VO_2_. This charging is very fast because of the low *R*_MET_ value. The voltage seen by the VO_2_ decreases until it reaches *V*_MIT_ and the transition from metal-to-insulator state occurs. These nano-oscillators are attractive for their area and potential energy efficiency.

**Figure 2:**
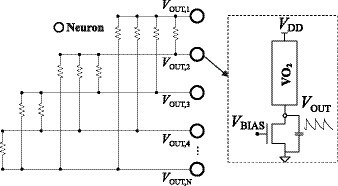
ONN design.

Figure 2 shows an ONN design using VO_2_-based nano-oscillators as neurons and resistive couplings as synapses (Corti et al., 2018, 2020). In this work it is shown that two resistively-coupled oscillators synchronize in phase when coupling strength is high enough (resistance value low enough) and in anti-phase for large enough resistance values. That is, they proposed to use resistive coupling for both positive and negative weights. However, it is not easy to select suitable resistance values in actual applications. In fact, capacitively coupling is the easiest way to achieve anti-phase synchronization. Both types of coupling can be implemented using only resistance or capacitances with differential oscillators. A differential VO_2_ oscillator has been proposed (Shamsi et al., 2021). It resorts to coupling two oscillators capacitively to force both outputs to be out of phase (180° apart). It allows for implementing both types of interactions using only capacitive or resistive coupling. This is very attractive from the point of view of implementing the coupling elements with memristor or ferroelectronic devices in crossbar architectures. On the basis of this oscillator, an ONN working as AM was shown by simulation (Shamsi et al., 2021).

In recent years, there have been numerous studies on ONNs using VO_2_. Most of them report simulation results. Although in some cases experimental results are also shown (Shukla et al., 2016; Corti et al., 2018, 2020; Dutta et al., 2019), they are not implementing the design techniques that other works on electrical simulations report that allow to improve the behavior of the ONNs (Shamsi et al., 2021). Therefore, experimental validation of these approximations is necessary. In this study, we describe an ONN realized in a commercial CMOS technology in which the oscillators are built using a circuit that we have developed to emulate the VO_2_ device. The purpose is to be able to study in-depth the synchronization dynamics of relaxation oscillators similar to those that can be performed with VO_2_ devices. The ONN has been designed to emulate not only VO_2_ devices but also fundamental characteristics of ONNs with this type of device, such as the fact that the interconnections between neurons are bidirectional. The fabricated circuit is very flexible since it allows programming the synapses to implement different ONNs, calibrating the frequency of the oscillators, or controlling their initialization. It uses differential oscillators and resistive synapses, equivalent to the use of memristors.

There are other two additional topics that must be introduced before proceeding with the CMOS ONN description.

The first one is the technique used to discretize the phase of the oscillators such that only two values are possible, and so the binary neurons of the reference HNN previously explained are reproduced. This can be achieved by Second Harmonic Injection Locking (SHIL) (Neogy and Roychowdhury, 2012). When a suitable synchronization signal, V_SYNC_, is injected into a non-linear oscillator, SHIL occurs, and the oscillator adopts a frequency half the frequency of V_SYNC_ (*f*_SYNC_) and becomes phase-synchronized within one of the two possible phases that are 180° apart. For this to occur, the natural frequency of the oscillator must be close to *f*_SYNC_/2. SHIL is also extremely useful to stabilize the oscillator frequency against variability effects, easing the oscillators to synchronize in frequency, which is required for proper operation of the ONN.

Finally, the AM operation requires the application of an input pattern to the ONN. This is equivalent to forcing a given ONN state (a given phase pattern). In Corti et al. (2018), a method for this is presented. The authors proposed forcing a given initial state by selectively delaying the supply voltage of each neuron. For example, assuming that only binary patterns are applied, such as black and white pixel images, the initial state of the network has only two different phases, 180° apart. Those oscillators corresponding to black pixels are in one phase, and those corresponding to white ones are in the other phase. To achieve this, the black oscillators are switched on at *T*_0_ and the white ones at *T*_0_ + *T*_OSC_/2, where *T*_OSC_ is the period of the oscillations.

The rest of the study is organized as follows. Section “Materials and Methods” describes in detail the designed and fabricated integrated circuit, along with the experimental setup prepared for testing it. Section 3 presents the experimental measurements and characterization of the circuit. Specifically, the ONN operation as an associative memory is demonstrated satisfactorily. The experiments carried out allow us to conclude that the ONN must be operated according to the type of computational task to be solved, and guidelines are extracted in this regard. Finally, section 4 summarizes the conclusions.

## 2 Materials and methods

### 2.1 Description of the fabricated CMOS differential ONN

An integrated circuit demonstrator of an analog 9-neuron ONN using a deep-submicron commercial CMOS technology (TSMC 65 nm – 1.2 V) has been designed, fabricated, and tested. The differential oscillators forming the neurons closely resemble those developed using VO_2_ devices and previously introduced. Oscillator frequencies can be calibrated. Couplings or synapses are implemented with a four-terminal six-transistor circuit, which conductive characteristics are determined by two voltages, allowing the implementation of positive and negative weights. The ONN is fully connected and programmable. For flexibility, it can operate both with and without SHIL. Operation of the fully differential ONN described in this section was extensively validated at post-layout level simulation.

**Figure 3:**
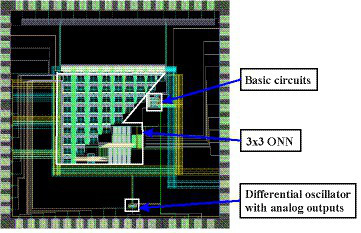
Layout of the fabricated circuit.

Figure 3 shows the layout of the fabricated circuit, showing the three types of circuits included (3×3 ONN, simple circuits, and differential oscillators with analog outputs) and their connections to the pad ring. The 3×3 ONN occupies a rectangle of 776 μm∙747 μm with some empty area inside, and the complete chip area, including pad ring, is 1710 μm∙1710 μm.

#### 2.1.1 Differential oscillator

**Figure 4:**
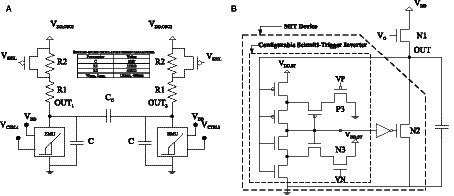
**(A)** Schematic of a differential oscillator. **(B)** Schematic of the circuit that emulates the behavior of the VO_2_ device.

Each neuron consists of a differential relaxation oscillator that is formed by two single-ended oscillators whose outputs (V_OUT1_ and V_OUT2_) are coupled by a capacitance (*C*_C_). In turn, each of the single-ended oscillators consists of a couple of resistors (*R1* and *R2*), a P transistor, a capacitance (*C*), and a CMOS circuit that emulates the voltage–current characteristic of a VO_2_, as shown in Figure 4A. The emulator (Figure 4B) has been designed using a Schmitt-Trigger inverter whose output is connected to a CMOS inverter that controls the gate voltage of an NMOS transistor (*N2*). Its drain and source terminals are the two terminals of the emulator. The input of the Schmitt-Trigger inverter is connected to the output of the oscillator. Unlike the conventional Schmitt-Trigger oscillator (Hodges and Jackson, 1983), in the proposed design, the output of the Schmitt-Trigger inverter is decoupled from the rest of the circuit, allowing its integration in complex oscillatory neural networks without penalty in energy efficiency by not having to increase its sizing. It also avoids using the floating resistor that appears in the conventional Schmitt-Trigger, whose implementation usually includes a switched capacitance and a switch. Additionally, the circuit includes the control functionality of the switching voltages *V*_IMT_ and *V*_MIT_, with the voltage *V*_N_ on the gate of transistor *N3*, providing the proposed solution with greater flexibility as it allows to make programmable both the frequency and the amplitude of the resulting oscillator.

**Figure 5:**
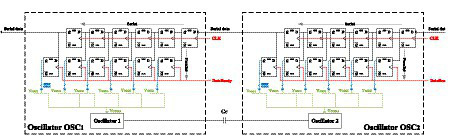
Schematic of oscillator calibration voltage selection based on series/parallel loading of the control word.

A step supply voltage is included for each single-ended oscillator (*V*_DD,OSC1_ and *V*_DD,OSC2_), through which the initial phase shift of the oscillator is controlled and, therefore, serves to establish the initial state of each neuron. Additionally, there are two inputs (*V*_CTRL1_ and *V*_CTRL2_), whose aim is to help with the frequency synchronization of the different oscillators in case it is compromised by the inherent variability of the process and mismatch. Each oscillator can be connected to any of the six available calibration voltages by means of programmable switches controlled by programming registers. This scheme is illustrated in Figure 5.

As previously mentioned, a general method to improve the stability and synchronization of oscillatory neural networks is using SHIL. In this circuit, SHIL is injected through PMOS transistors, which are turned on and off through the input signal *V*_SHIL_.

#### 2.1.2 Synapse

**Figure 6:**
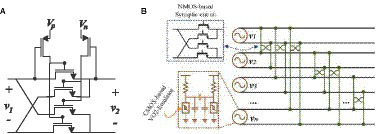
**(A)** Schematic of the synapsis. **(B)** Differential ONN implementation from Shamsi et al. (2021).

As an analogy of the Wheatstone bridge, Figure 6A shows the schematic of the synaptic circuit, which is capable of providing positive, negative, and zero weights. Being a four-terminal circuit makes it appropriate for differential structures. Depending on the gate voltages, it is possible to have positive (*V*_P_ > *V*_N_), negative (*V*_N_ > *V*_P_), or zero weight (*V*_P_=*V*_N_). The two PMOS transistors are used for controlling the current between the neurons. Transistors between the positive branches can transfer current when (*V*_P_*-V*_1_*^+^*) *< V*_TH_ because *V*_P_ is applied to their gate. In addition, transistors between the negative branches can transfer current when (*V*_N_*-V*_2_*^+^*) *< V*_TH_ because *V*_N_ is applied to their gate. Therefore, the current between the neurons is controlled using these PMOS transistors. Figure 6B depicts the topology of the fabricated differential ONN.

A training rule is used to store patterns in neural networks, adjusting the synaptic weights accordingly. Once the weights are known, we propose a mapping rule to obtain the physical resistances for the memristor-bridge synapses. To store patterns in the ONN, we use the Hebbian rule to calculate the weights:


(2) $$W_{ij}=\frac{1}{L}\overset{P}{\underset{k=1}{\sum }}b_{i}^{k}b_{j}^{k}\;i,j\in \left\{1,2,3,\ldots ,L\right\}$$

where *P* is the number of stored patterns and *L* is the number of pixels in each pattern (which is equal to the number of neurons in the ONN). Elements *b*_i_ and *b*_j_ of all stored patterns are used to calculate the weight *W*_ij_.

We propose here the following rules to map the sign and value of the above weights to the controlling voltages *V*_P_ and *V*_P_. Weights *W*_ij_ are mapped to the *V*_P_ and *V*_P_ values using the following relation:


(3) $$\mathrm{Map}:\{\begin{array}{c} \alpha V_{P}=V_{N}=V_{0}\;w_{ij}<0\\ \begin{array}{c} V_{P}=\alpha V_{N}=V_{0}\;w_{ij}>0\\ V_{N}=V_{N}=V_{0}\;w_{ij}=0 \end{array} \end{array}$$

where α>1 is a constant value. The design parameters of the synaptic circuit are *W*_ij_, *α*, and *V*_0_. Parameters *α* and *V*_0_ will be obtained based on correctly functioning hardware.

Each synapse control voltage can be connected selectively to different voltages using programmable switches controlled by programming registers, similar to the oscillator calibration shown in Figure 5.

### 2.2 ASIC description

The ASIC consists of the following blocks:

– ONN of nine differential oscillators is fully interconnected with each other through 36 synapses. The ONN includes a control system from which the voltages defining the synapse weights can be selected, as well as an oscillator calibration mechanism to improve network synchronization. The oscillator outputs are connected to digital pads through Schmitt-Trigger buffers with configurable hysteresis to regenerate rail-to-rail signal swings and to cope with the waveform shape of relaxation oscillators.
– Basic circuits. Specifically, a single-ended oscillator, a differential oscillator, and two differential oscillators connected through a synapse similar to the one used in the ONN have been included. In all of them, the output is digital, as in the ONN. In addition, a differential oscillator has been included whose outputs are connected directly to analog pads in order to be able to observe the waveforms without digitizing.

#### 2.2.1 Description of key signals/pads

The signals involved in the circuit are divided into the following categories:

– Oscillator supply voltages: Since differential oscillators are being used and there are nine oscillators in the ONN, 18 signals are required. These signals are generated externally and applied to digital pads that generate step signals between 0 V and 1.2 V. Controlling the relative timing on the initial phase selection step is essential: a delay between both signals corresponding to half a period involves applying input stimuli with opposite phases.
– Control system signals: Includes the clock signal, the signal that codifies the information to be loaded into the calibration/programming voltage selection registers, and the signal that indicates that the information has been loaded into the serial registers and serial-to-parallel conversion can be done.
– Oscillator calibration signals: These six signals can take values between 0 V and 1.2 V and allow the oscillator frequency to be tuned.
– Synapse programming signals: These 12 signals are used to set the weights for the synapses. They take values between 0 V and 1.2 V.
– Output stage configuration signals: These signals are used to set the thresholds of the ONN output Schmitt-Trigger buffer.
– Synchronization signal: It is a digital signal that ranges between 0 V and 1.2 V, with a frequency double that of the ONN’s oscillators, used to enable the SHIL mechanism.

#### 2.2.2 Control logic for calibration and programming

Each oscillator and each synapse have twice as many flip-flops as switches to be controlled. That is, 12 for each oscillator (see Figure 5) and 12 for each synapse. Half of them are configured in a single shift register, generating, therefore, a connection of 12∙9 + 12∙36 = 540 memory elements. These registers are controlled by the clock signal. A control word is serially loaded in the shift register. It contains the calibrating and programming bits indicating which switches are closed and which are not. Obviously, for each oscillator or synapse, only one of its switches should be closed. Once the control word is fully loaded, a signal that indicates that the data are ready to be loaded is activated, and the information contained in the shift registers is loaded in parallel to the flip-flops directly controlling the switches.

### 2.3 Test board and experimental setup

**Figure 7:**
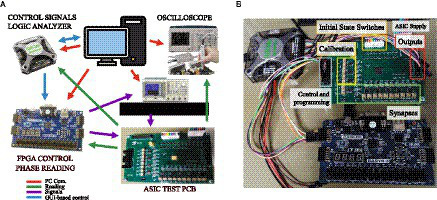
**(A)** Block diagram and **(B)** photograph of the test setup.

The experimental verification of the ASIC has been performed using a custom-designed test PCB for this purpose. The block diagram of the setup and test equipment is shown in Figure 7A, together with a photograph of the test PCB and its wiring in Figure 7B. The FPGA is used for programming the ONN (providing the bitstream that configures the assignment of synapses and calibration voltages) and for controlling the time-delayed power-on of the differential oscillators. A discrete micro-switch on-board allows to configure the initial state of the system, on which basis input patterns are applied to the ONN.

The main features of the PCB are summarized hereafter. First, the initialization of the differential oscillators is performed using two digital signals (common for each of them), which abruptly commute from low to high level with a delay equivalent to half an oscillator period. These signals are applied as the supply voltages of the differential oscillators. The order in which they are applied is given by the position of the micro-switch associated with each oscillator (labeled in Figure 7B as ‘Initial State Switches’). These signals are generated and switched off by the custom digital design in the FPGA, commanded by START and RESET signals sent by the Digital Discovery instrument. A commercial software application linked to the instrument allows the PC user to trigger these signals.

In addition, the synchronization signal for SHIL is provided using the Tektronix AFG3102 function generator, whose output is connected to the SMA connector on the PCB, which has access to an ASIC’s digital pad. Typically, the waveform used for the synchronization signal is a square signal with a voltage range between 0 V and 3.3 V. It is controlled by a trigger signal generated from the FPGA, allowing for the control of the time scheduling of both the start of the synchronization signal and the initialization of the oscillators.

The generation of the calibration and programming voltages is carried out on-board with a simple circuit consisting of an operational amplifier, a potentiometer, resistors, and capacitors. Each of the 12 programming and the six calibration voltages has one instance of this circuit dedicated, with an individual potentiometer, as can be seen in the ‘Calibration’ and ‘Synapses’ boxes in Figure 7B.

Finally, regarding output observation, digital ones are monitored using oscilloscope probes or the logic analyzer included in the Digilent Digital Discovery instrument. Furthermore, the FPGA I/O pins are compatible with reading it directly. Analog outputs can be observed using oscilloscope probes. A Keysight DSOX4104A oscilloscope has been used.

## 3 Results

### 3.1 Exploring the dynamics

The first aim of the ASIC was to be able to explore the dynamics of coupled oscillator systems. Thus, before describing its application to solve computation tasks, we report on the results of a set of experiments carried out to analyze the behavior of neurons, synapses, and the SHIL mechanism.

#### 3.1.1 Oscillator performance

**Figure 8:**
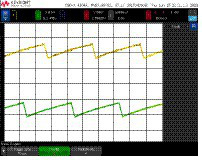
Experimental waveforms for a differential oscillator with analog outputs.

Although the ONN system has only digital outputs, simple analog oscillators were also included in the chip and connected to analog pads in order to be able to observe their behavior. Figure 8 depicts the experimental waveforms for an analog differential oscillator identical to the ones in the ONN. As expected, both outputs are 180° apart. The output average voltage ranges from 379 mV to 763 mV. Note that the small oscillation amplitude justifies the carefully designed Schmitt-Trigger-based output stage included for digitalization.

**Figure 9:**
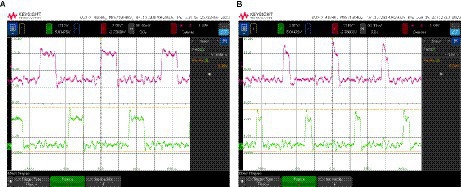
Experimental waveforms corresponding to the outputs of one of the oscillators after digitalization applying two calibration voltages: **(A)** 1.2 V and **(B)** 0.9 V.

Figure 9 depicts the two outputs of one of the oscillators after digitalization and applying two calibration voltages. Figure 9A is for 1.2 V, where it can be observed that the outputs are out of phase, showing correct operation. The waveforms in Figure 9B correspond to the same experiment for a calibration voltage of 0.9 V. Note the frequency differences: by reducing the voltage applied to the calibration input, the frequency increases. The measured frequencies are 5.9 MHz and 7.18 MHz, respectively. The nine ONN oscillators have been characterized with a calibration voltage of 1.2 V. The average frequency obtained ranges between 5.5 MHz and 5.9 MHz, with a relative standard deviation between 25 m and 8 m. By varying the calibration voltage, it is possible to individually tune the frequencies of each oscillator between 6.02 MHz and 6.12 MHz, leading to a frequency difference reduction of a factor of 4.

**Figure 10:**
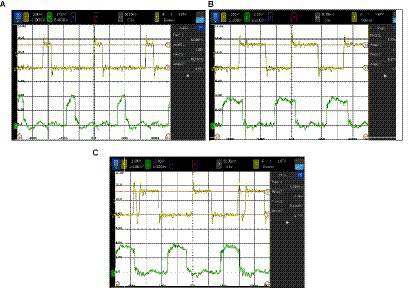
Impact of the configuration of the output buffer stage on the duty cycle of the output voltage. **(A, B)** show how the duty cycle of the output voltage varies when the output buffer configuration is modified. **(C)** shows that an undesired glitch may appear if the setting is not correct.

Figure 10 depicts the obtained waveforms for the positive output of oscillator 1 with 1.2 V in the calibration voltages for different configurations of the output buffer stage. It can be observed that the duty cycle of the digital oscillator changes. In Figure 10C, control voltages have been selected so that an undesired glitch is observed.

All these experiments have been carried out with deactivated SHIL. This is achieved by applying a constant of 3.3 V to the SHIL signal PAD.

#### 3.1.2 Second harmonic injection locking performance

A common SHIL signal, externally provided, enables the synchrony between the oscillators when the SHIL frequency is found in a determined range related to the natural oscillator frequency. To illustrate the impact of applying SHIL, the average frequency and the deviation with and without SHIL were measured. With SHIL at 13.9 MHz, the average frequency increases to 6.95 MHz, and the relative deviation reduces to 4 m. Note that the oscillator synchronizes to half the SHIL signal as expected, and the SHIL signal helps to reduce the impact of oscillator jitter on frequency variation.

**Figure 11:**
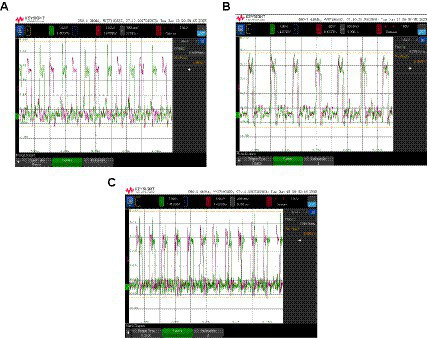
Output of two uncoupled oscillators is depicted with and without SHIL. **(A)** and **(B)** with SHIL and **(C)** without SHIL.

It is well-known that SHIL discretizes the oscillator phase so that only two phases can occur. These two phases are ideally 180° apart. This is shown in Figure 11. The output of two uncoupled oscillators is depicted with and without SHIL. Figures 11A,B corresponds to the case with SHIL. Since each oscillator can be in one of the two phases, they can both be either in-phase or anti-phase. We have been able to capture the two behaviors by slightly modifying the SHIL frequency. Note that the frequencies of the signals in Figures 11A,B are very close (6.769 MHz and 6.778 MHz), indicating that the modification of the SHIL signal has been minimal and yet able to introduce noise that leads to an oscillator being able to jump from one phase to the other. In no case has a situation been observed where the two oscillators have a phase difference other than 0° or 180°. By deactivating SHIL, any phase difference is possible (Figure 11C). Note that in these experiments, the two oscillators were individually calibrated to equalize their frequencies.

#### 3.1.3 Synapse performance

**Figure 12:**
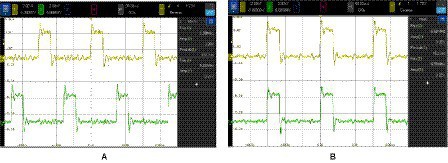
Two coupled oscillators in which the synapse is programmed with **(A)** negative coupling: *V*_P_ = 0 V and *V*_N_ = 0.95 V and **(B)** positive coupling: *V*_P_ = 0.95 V and *V*_N_ = 0 V.

As it was described, the implemented synapse can be programmed to enable both a positive and a negative coupling between a pair of oscillators. Positive (negative) coupling forces the two oscillators to be in phase (out of phase). Figure 12 shows the two cases. In Figure 12A, the synapse connecting the two oscillators was programmed with *V*_P_ = 0 V and *V*_N_ = 0.95 V. Figure 12B corresponds to *V*_P_ = 0.95 V and *V*_N_ = 0 V. The depicted waveforms have been obtained with SHIL activated. In this condition, the range of synapse voltages for which coupling is achieved is wide (from 0.25 V to 1.2 V). However, when there was no SHIL, this range was significantly reduced. The minimum required voltage increases to 0.95 V. Additionally, the range of valid SHIL frequencies is reduced with the synapse voltage. That is, both SHIL and coupling strength contribute to the operation of coupled oscillator systems.

**Figure 13:**
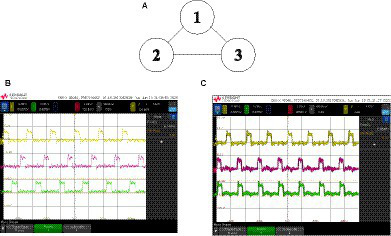
**(A)** Three coupled oscillators with an all-to-all connectivity. Output waveforms: **(B)** when SHIL is not applied and **(C)** when SHIL is applied.

To finish this first section on experimental results, we describe the behavior of three coupled oscillators with all-to-all connectivity. That is, each of the three differential oscillators is coupled, as depicted in Figure 13A. The type of coupling is negative, and so the phases of each pair of oscillators are forced to separate from each other. We have just shown that a pair of negative-coupled oscillators evolved toward the anti-phase relationship. Clearly, when there are three connected oscillators, as in Figure 13A, it is not possible to satisfy that relationship for every pair of oscillators. It is not possible that O1 is in anti-phase with O2 and with O3, and, at the same time, O2 and O3 are also in anti-phase. It is interesting to check that our system behaves as expected. This expected behavior is completely different whether SHIL is applied or not. Assuming identical coupling, when no SHIL is applied, the three phases tend to be equally distributed (ideally to be 120° apart from each other). This is the state of the network that minimizes energy. With SHIL, since the phases are binarized, such a phase pattern is not allowed. The system tends to satisfy as many anti-phase relationships as possible. In this case, two out of the three can be satisfied.

The waveforms we experimentally obtained are depicted in Figure 13B when no SHIL was applied and in Figure 13C when SHIL was applied. The three synapses were programmed identically with *V*_P_ = 0 V and *V*_N_ = 0.8 V. It can be observed that the expected behavior is obtained. Note that without SHIL, the three oscillators are not exactly 120° apart in phase. This can be due to variability in synapses, so that although the applied voltages are identical, the coupling strength can be slightly different.

It is interesting to point out that in these examples, we are in fact using physics to solve well-known computation problems. Without SHIL, the system solves the graph coloring problem (Wu et al., 2011; Parihar et al., 2017) associated with the triangle in Figure 13A. Different phases mean different colors for the nodes associated with the oscillators. With SHIL, the system obtains the Max-Cut of the corresponding graph. Nodes are split into two sets such that the number of edges between both groups is the maximum. A cut value of 2 was obtained in this case. Max-Cut is just one example of a problem that can be solved by coupled oscillator systems. A great interest has recently aroused in implementing oscillator-based Ising Machines (OIMs). OIMs efficiently solve Ising models, and there are procedures to map many hard-combinational problems into Ising models (Lucas, 2014).

### 3.2 ONN as associative memory

As it was described in the introductory section, an ONN can be used as an AM useful for pattern recognition applications. Unlike the graph coloring or Ising solver functionalities of the ONN described in the previous sub-section, the AM operation required applying an input pattern to the ONN. Thus, the initial phase pattern in the oscillators needs to be controlled in order to represent the input information. Corti et al. (2018) proposed to do it by controlling the timing of the power-on of each oscillator. Different works have shown that AM functionality can be achieved with this method (Núñez et al., 2021; Shamsi et al., 2021). In order to be able to use this initialization mechanism, the supply voltage of each oscillator can be independently controlled in our design, as previously described. Thus, we can test the operation of the fabricated ONN as an AM.

**Figure 14:**
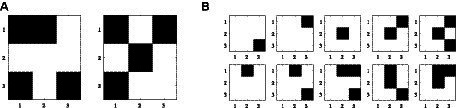
**(A)** Two stored patterns and **(B)** 10 test patterns selected for the measured 3×3 ONN AM.

Figure 14A depicts the two selected patterns to be stored, and Figure 14B shows the distorted test patterns applied to validate the AM operation. Note: We used binary patterns representing a black and white 3×3 image to approximate the pattern recognition application.

The required coupling type (positive or negative) and strength were determined for each synapse from the weight matrix obtained with Hebb’s rule, and so the ONN was programmed accordingly. There were three different coupling values (one positive, one negative, and one null for uncoupling). Therefore, only two different voltages were required for biasing the synaptic circuit. Positive (negative) couplings were coded with *V*_P_ = 0.85 V and *V*_N_ = 0 V (*V*_P_ = 0 V and *V*_N_ = 0.85 V) and uncoupled with *V*_P_=*V*_N_ = 0 V. A SHIL signal of 14.3 MHz was injected.

Table 1 reports the obtained results for the two stored patterns (P1 and P2) and the 10 test patterns (T1–T10) that had been used during design for post-layout validation. For each, we indicate:

- Hamming distance to the closest store pattern (in parenthesis).
- The expected pattern is to be retrieved according to the associative memory functionality. For each test pattern, we expect to retrieve the closest stored one in terms of Hamming distance. This distance metric is the number of elements that are different between two patterns.
- Experimentally retrieved (read) pattern. A total of 100 trials were carried out for each input pattern. The number of times P1 is retrieved, followed by the number of times P2 is retrieved, is depicted. The reading operation is carried out at different time instants after the application of the test patterns, concretely at 3, 10, and 720 oscillation cycles from the beginning. Considering that the oscillation period is approximately 140 ns, these measurements correspond to 42 ns, 1.4 μs, and 100.5 μs.

**Table 1:** Summary of experimental results corresponding to the associative memory.

	Expected	Read (@ 3 cycles)	Read (@ 10 cycles)	Read (@ 720 cycles)
P1	P1	100/0	100/0	100/0
P2	P2	0/100	0/100	0/100
T1 (3)	P1	100/0	100/0	100/0
T2 (3)	P2	0/100	0/100	0/100
T3 (3)	P2	0/100	0/100	0/100
T4 (2)	P2	0/10	0/100	0/100
T5 (3)	P2	0/100	0/100	0/100
T6 (3)	P1	100/0	100/0	100/0
T7 (2)	P1	100/0	100/0	100/0
T8 (3)	P1	100/0	100/0	100/0
T9 (3)	P1	100/0	100/0	100/0
T10 (3)	P2	0/100	0/100	0/100

The test patterns were evaluated with the mathematical HNN model obtaining the expected pattern for all of them, as well as the ONN does. Particularly, T4 is the only test pattern that did not converge the expected pattern in 90 out of 100 at the first reading at 3 cycles, but it quickly inferred and stabilized in the correct pattern from the second reading at 10 cycles, 1 μs later. Additionally, it can be observed from the third read at 720 cycles that the retrieved pattern is kept. So, it is concluded that the ONN successfully stores the two patterns. In fact, the only two stable states that were observed in all our experiments are those patterns. It has also been demonstrated that the ONN exhibits associative memory functionality. That is, it is able to retrieve a stored pattern from an applied pattern that is not a stored one.

## 4 Discussion

A 9-neuron CMOS ONN resembling a VO_2_-based ONN has been designed, fabricated, and tested. It uses a CMOS sub-circuit emulating the I–V characteristic of VO_2_ devices to build differential oscillators. The synapse is implemented with a 6-transistor bridge topology, enabling resistive coupling among oscillators. Both positive and negative weights can be realized. The fabricated ASIC is programmable, with a large degree of controllability and observability to be able to dive into the dynamics of coupled non-linear oscillators, on which basis the computation is carried out.

Experiments carried out with two coupled oscillators (sub-section 3.1.3) have allowed us to experimentally sustain that both SHIL and coupling strength contribute to the synchronization of the oscillators.

The AM functionality has been demonstrated. It is important to point out that when we started to test the AM functionality, the results were not completely deterministic. That is, repeating an experiment several times led to different results. For some of the test patterns, sometimes P1 was retrieved, while for others it was P2. After carefully analyzing this behavior, we noted that conditions were not actually identical across the 100 trials since the SHIL signal was continuously running. So, the timing of oscillators power-on with respect to the SHIL phase was not fixed. We solved it by synchronizing the SHIL signal triggering and the initialization of the oscillators. Even after this modification of the experimental setup, there was still some indeterministic behavior in the system associated with input patterns equidistant (in terms of Hamming distance) to the two stored patterns. For those input patterns, sometimes P1 was retrieved while others were P2. It was due to the impact of noise.

Furthermore, it was observed that the system could evolve from one stable state to another. For example, as described in sub-section 3.1.2, a slight frequency shift of the SHIL induces noise that triggers a phase shift of an oscillator. Moreover, without any intended action on the experimental setup, and due to internal noise and other non-controllable noise sources, the phase flip can occur. It is extremely important to point out that the rate of this event is very different whether SHIL is applied or not. Under SHIL, this rate is much lower. In fact, we were not able to observe jumps from P1 to P2 in the AM with SHIL, although they occurred if SHIL was deactivated.

This observed behavior is very interesting from the point of view of the application of ONNs as Ising machines. The OIM application requires being able to escape from local energy minima. Our findings illustrate that scaping is easier in the absence of SHIL and that it can be enhanced by noise. This agrees with different works stating the importance of the SHIL signal schedule to improve the probability of exactly solving the associated Ising model. That is, obtaining a phase distribution corresponding to the minimum configuration of the Ising Hamiltonian. The next step in the exploitation of this integrated circuit is linked to the validation of the results reported in Avedillo et al. (2023), related to the resolution of combinatorial optimization problems.

## Data availability statement

The raw data supporting the conclusions of this article will be made available by the authors, without undue reservation.

## Author contributions

MJ: Writing – original draft, Writing – review & editing. JN: Writing – original draft, Writing – review & editing. JS: Writing – original draft, Writing – review & editing. BL-B: Writing – review & editing. MA: Writing – original draft, Writing – review & editing.
